# Effects of Taurine in Mice and Zebrafish Behavioral Assays With Translational Relevance to Schizophrenia

**DOI:** 10.1093/ijnp/pyac073

**Published:** 2022-10-14

**Authors:** Franciele Kich Giongo, Matheus Gallas-Lopes, Radharani Benvenutti, Adrieli Sachett, Leonardo Marensi Bastos, Adriane Ribeiro Rosa, Ana Paula Herrmann

**Affiliations:** Laboratório de Neurobiologia e Psicofarmacologia Experimental (PsychoLab), Departamento de Farmacologia; Programa de Pós-Graduação em Farmacologia e Terapêutica; Laboratório de Neurobiologia e Psicofarmacologia Experimental (PsychoLab), Departamento de Farmacologia; Programa de Pós-Graduação em Neurociências; Programa de Pós-Graduação em Neurociências; Laboratório de Neurobiologia e Psicofarmacologia Experimental (PsychoLab), Departamento de Farmacologia; Programa de Pós-Graduação em Farmacologia e Terapêutica; Laboratório de Psiquiatria Molecular, Hospital de Clínicas de Porto Alegre, Porto Alegre, Rio Grande do Sul, Brazil; Laboratório de Neurobiologia e Psicofarmacologia Experimental (PsychoLab), Departamento de Farmacologia; Programa de Pós-Graduação em Farmacologia e Terapêutica

**Keywords:** behavior, C57BL/6, MK-801, schizophrenia, taurine, zebrafish

## Abstract

**Background:**

Altered redox state and developmental abnormalities in glutamatergic and GABAergic transmission during development are linked to the behavioral changes associated with schizophrenia. As an amino acid that exerts antioxidant and inhibitory actions in the brain, taurine is a potential candidate to modulate biological targets relevant to this disorder. Here, we investigated in mice and zebrafish assays whether taurine prevents the behavioral changes induced by acute administration of MK-801 (dizocilpine), a glutamate N-methyl-D-aspartate (NMDA) receptor antagonist.

**Methods:**

C57BL/6 mice were i.p. administered with saline or taurine (50, 100, and 200 mg/kg) followed by MK-801 (0.15 mg/kg). Locomotor activity, social interaction, and prepulse inhibition of the acoustic startle reflex were then assessed in different sets of animals. Zebrafish were exposed to tank water or taurine (42, 150, and 400 mg/L) followed by MK-801 (5 µM); social preference and locomotor activity were evaluated in the same test.

**Results:**

MK-801 induced hyperlocomotion and disrupted sensorimotor gating in mice; in zebrafish, it reduced sociability and increased locomotion. Taurine was mostly devoid of effects and did not counteract NMDA antagonism in mice or zebrafish.

**Discussion:**

Contradicting previous clinical and preclinical data, taurine did not show antipsychotic-like effects in the present study. However, it still warrants consideration as a preventive intervention in animal models relevant to the prodromal phase of schizophrenia; further studies are thus necessary to evaluate whether and how taurine might benefit patients.

**Significance Statement**

Antipsychotic drugs are the main pharmacological strategy to manage the symptoms of patients with schizophrenia. However, novel therapeutic options are needed because currently available drugs do not deliver a full recovery and many patients are unresponsive to treatment. Taurine is an amino acid that exerts modulatory actions in the brain with potential relevance to schizophrenia. Here, we investigated taurine properties in preclinical behavioral experiments using mice and zebrafish. We found that taurine was mostly devoid of effects and did not counteract the behavioral alterations induced in such animal models. Our results contradict previous clinical and preclinical data, but further studies are necessary to evaluate whether and how taurine might benefit patients.

## INTRODUCTION

Schizophrenia is a serious mental illness that remains one of the main challenges in modern psychiatry. With an incidence slightly higher in men than women (Jongsma et al., 2019), schizophrenia affects approximately 1 in 300 people (World Health Organization, 2022) and dramatically changes the individual’s life course. Psychotic symptoms, social isolation, cognitive impairment, and stigma are only a few of the obstacles that contribute to the poor prognosis of this condition. Hyperdopaminergic activity in subcortical areas is associated with the onset of psychotic symptoms (McCutcheon et al., 2018) and might be linked to the loss of fast-spiking parvalbumin-positive GABAergic interneurons, hypofunction of glutamate NMDA receptors, and oxidative stress (Cabungcal et al., 2013; Grace, 2016; Hardingham and Do, 2016). Furthermore, several studies have indicated increased frequency of smoking (Lasser et al., 2000), alcohol or illegal substances misuse (Hjorthøj et al., 2015), sedentary lifestyle (Stubbs et al., 2016), and poor dietary habits (Dipasquale et al., 2013) among individuals with schizophrenia, which results in a 13- to 15-year reduction in life expectancy, currently averaged at 65 years (Hjorthøj et al., 2017).

Early and continuous administration of antipsychotic drugs is the main pharmacological strategy; unfortunately, however, the currently available drugs do not provide a full recovery, and one-third of the patients are unresponsive to treatment (Jääskeläinen et al., 2013; Bruijnzeel et al., 2014). Antipsychotic drugs, which act by blocking dopamine D_2_ receptors, ameliorate mainly the positive symptoms (e.g., hallucinations and delusions), with little to no effect on negative symptoms (e.g., social isolation, depression, avolition) and cognitive impairment (e.g., poor long-term memory, sustained attention, and cognitive performance) (Shafer and Dazzi, 2019). Pharmacological interventions pose yet another challenge: side effects such as extrapyramidal symptoms, metabolic syndrome, and weight gain further impact quality of life and compromise adherence to treatment (Ellenbroek, 2012; Bruijnzeel et al., 2014).

Since the observation that phencyclidine induces in healthy individuals a psychotic state that resembles schizophrenia (Luisada and Brown, 1976; Allen and Young, 1978), NMDA antagonists have been used to recapitulate relevant behavioral alterations in animal models (Jones et al., 2011). Other non-competitive NMDA antagonists, such as MK-801 (dizocilpine) and ketamine, also trigger complex effects similar to the positive, negative, and cognitive symptoms experienced by individuals with schizophrenia (Buffalo et al., 1994; Adler et al., 1999; Bondi et al., 2012).

Mice and rats are the most commonly used species to study schizophrenia-relevant behavior in animals due to their high degree of homology with human physiology, neurochemistry, brain morphology, and circuitry (Meyer and Feldon, 2010). More recently, zebrafish emerged as another popular vertebrate model animal in biomedical research. The advantages of using zebrafish include their high fertility and fast development, which makes this species a less expensive and space-demanding option of laboratory animal (Khan et al., 2017) while also having a neural architecture highly comparable with mammals (Parker et al., 2013). However, a clear limitation of any model animal is the impossibility to recapitulate the verbal components of schizophrenia symptoms; we thus have to rely on non-verbal phenotypes, like hyperlocomotion or stereotypy, as proxies of schizophrenia-like behavior (Tordjman et al., 2007). As the complexity of mental disorders further widens the gap between animals and humans, using multiple model organisms has the potential to improve translatability due to increased external validity.

Here, we used acute administration of MK-801 in mice and zebrafish to study the antipsychotic-like properties of taurine in behavioral assays with translational relevance to schizophrenia. Taurine, also known as 2-aminoethanesulfonic acid, is the most abundant free amino acid in the human body (Huxtable, 1992) and acts as an inhibitory neuromodulator in the brain (Oja and Saransaari, 1996); it also has neuroprotective, antioxidant, and immunomodulatory properties (Almarghini et al., 1991; Redmond et al., 1998). The effects of taurine in the central nervous system are likely mediated by agonism at GABAergic and glycinergic receptors, yet it is still unknown whether taurine-specific receptors could be involved (Oja and Saransaari, 2015). Several studies have shown altered levels of taurine in the brain and plasma of schizophrenia patients and in animal models. Increased taurine levels were observed in the prefrontal cortex of schizophrenia patients as well as a correlation between increased taurine and disease duration (Shirayama et al., 2010). A recent study also observed elevated taurine levels in serum samples of first psychotic episode and early-stage patients (Parksepp et al., 2020). In contrast, decreased taurine levels were observed in the cerebrospinal fluid of drug-naïve individuals (Do et al., 1995). In a mouse model of prenatal immune activation, taurine was decreased in the hippocampus, striatum, and temporal and parietal cortex (Winter et al., 2009; Yang et al., 2019). Finally, a recent double-blind randomized trial on 86 individuals with first-episode psychosis showed a significant improvement in schizophrenia symptoms in patients who received taurine as an adjuvant treatment compared with placebo (O’Donnell et al., 2016), and preclinical zebrafish data further support the potential benefits of taurine in this context (Franscescon et al., 2020, 2021).

Considering the above-cited evidence, this study aimed to test the hypothesis that acute administration of taurine prevents schizophrenia-relevant behavioral alterations induced by acute administration of MK-801 in mice and zebrafish assays.

## METHODS

### Animals

#### Mice

C57BL/6 male mice (7–14 weeks old, 20–30 g) were obtained from an external vendor (Centro de Cardiologia Experimental—Instituto de Cardiologia, Porto Alegre, Brazil). Upon arrival at Unidade de Experimentação Animal (Hospital de Clínicas de Porto Alegre), animals were housed in groups of 3 to 5 animals per cage (20 × 30 × 13 cm) for at least 2 weeks before experiments. Different sets of animals were used for each of the behavioral assays. The animals were maintained under controlled environmental conditions (reversed 12-h-light/-dark cycle with lights on at 7:00 am and constant temperature of 22°C  ± 1°C) with free access to food (Nuvilab CR-1, Colombo, Brazil) and water. All procedures were approved by the animal welfare and ethical review committee of Hospital de Clínicas de Porto Alegre (approval no. 180498) and were performed in accordance with the relevant guidelines on care and use of laboratory animals and the Brazilian legislation.

#### Zebrafish

Experiments were performed using male and female (50:50 ratio) short-fin wild-type zebrafish (6 months old, 400–500 mg). Because zebrafish reach sexual maturity at approximately 3 months post fertilization, we used adult animals of a developmental stage comparable with the mice used in this study (Singleman and Holtzman, 2014). Animals were obtained from a local commercial supplier (Delphis, Porto Alegre, Brazil). The animals were maintained at Instituto de Ciências Básicas da Saúde in a light/dark cycle of 14/10 hours with lights on at 7:00 am for at least 2 weeks before tests. Fish were kept in 16-L (40 × 20 × 24 cm) unenriched glass tanks with non-chlorinated water at a maximum density of 2 animals per liter. Tank water satisfied the controlled conditions required for zebrafish (26°C ± 2°C; pH 7.0 ± 0.3; dissolved oxygen at 7.0 ± 0.4 mg/L; total ammonia at <0.01 mg/L; total hardness at 5.8 mg/L; alkalinity at 22 mg/L CaCO_3_; conductivity of 1500–1600 μS/cm) and was constantly filtered by mechanical, biological, and chemical filtration systems (Altamar, Jacareí, Brazil). Food was provided twice a day as commercial flake food (Poytara, Araraquara, Brazil) plus brine shrimp (*Artemia salina*). The sex of the animals was confirmed after killing by dissecting and analyzing the gonads. Animals were killed by hypothermic shock according to the AVMA Guidelines for the Euthanasia of Animals (Leary and Johnson, 2020). For all experiments, no sex effects were observed, so data were pooled together. All procedures were approved by the animal welfare and ethical review committee at the Universidade Federal do Rio Grande do Sul (approval no. 35525).

### Drugs

Taurine and MK-801 (dizocilpine) were purchased from Sigma-Aldrich (St. Louis, MO, USA). For rodent experiments, drugs were dissolved in saline (0.9% NaCl), and solutions were freshly prepared and injected i.p. at a volume of 5 mL/kg. Animals were manually contained for drug administration. For the zebrafish assay, MK-801 and taurine were dissolved in tank water; solutions were freshly prepared and renovated halfway through the experiment.

The dose of MK-801 for mice experiments was based on previous reports (Wu et al., 2005; Meyer et al., 2008; Suryavanshi et al., 2014; Giovanoli et al., 2016) and pilot experiments; the concentration of MK-801 for the zebrafish assay was based on Benvenutti et al. (2022). Taurine doses for mice experiments were defined as a dose curve in logarithmic scale covering a range reported in the literature (Wu et al., 2017; Caletti et al., 2018); taurine concentrations used in the zebrafish assay were the same employed by Franscescon et al. (2020, 2021) and other studies by the same group.

### Experimental Design

**Figure 1.**
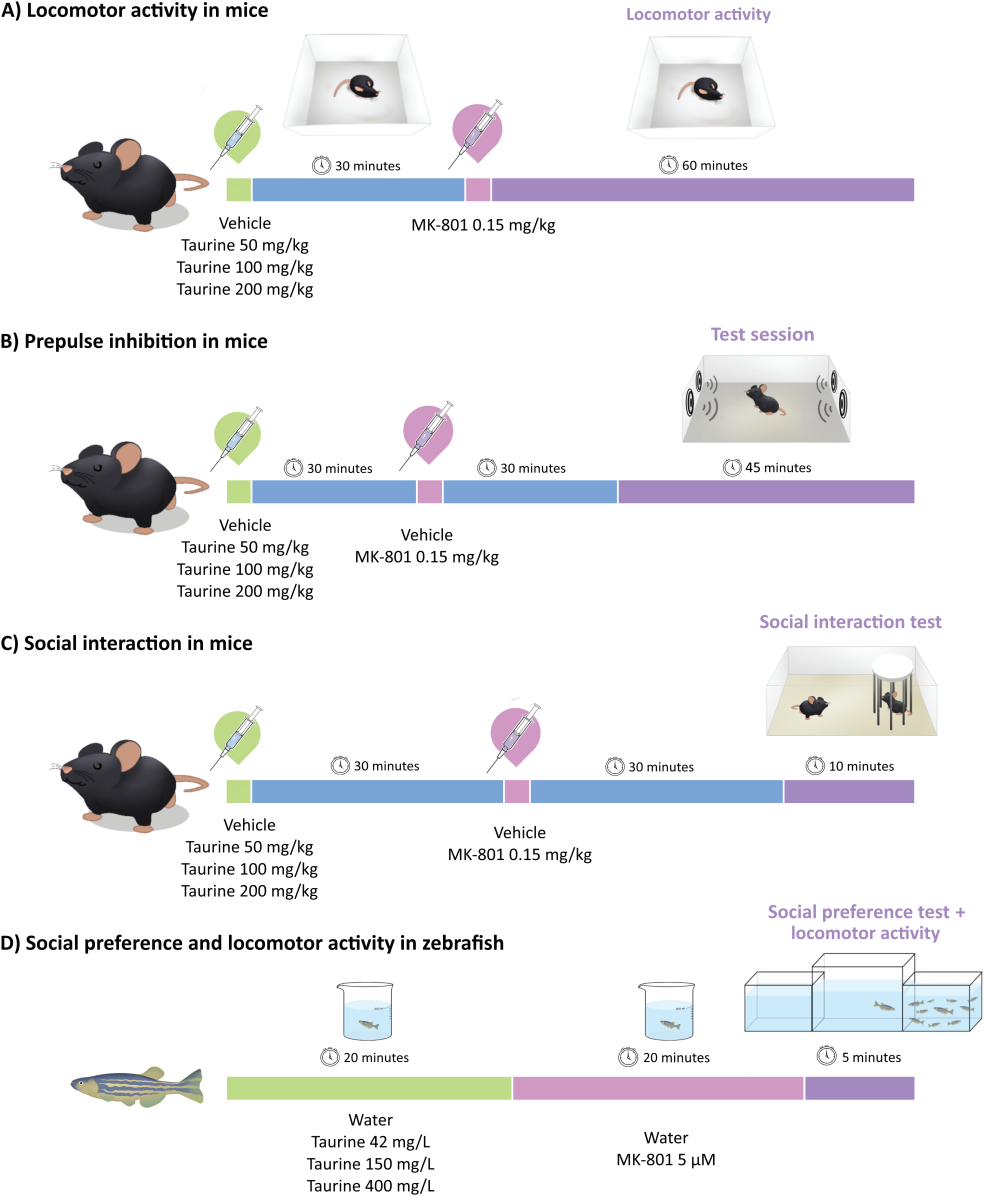
Overview of the experimental design. (A) Locomotor activity in mice, (B) prepulse inhibition of the startle reflex in mice, (C) social interaction in mice, (D) social preference and locomotor activity in zebrafish.

Different sets of animals were used for each experiment, totaling 288 mice and 96 zebrafish in the study. The animals were allocated to the experimental groups following block randomization procedures to counterbalance for litter and cage in mice experiments and for sex and home tank in zebrafish experiments. The order for outcome assessment was also randomized, and care was taken to counterbalance the test apparatuses across the experimental groups. Outcome assessors were blind to the experimental groups as well as the experimenters responsible for taking the animal and placing it in the test apparatus. An overview of the experimental design is illustrated in Figure 1.

We report how we determined our sample size, all data exclusions, all manipulations, and all measures in the study. Raw data and analyses outputs were deposited in the Open Science Framework and are openly available at https://osf.io/qy2uw (Giongo et al., 2022).

#### Locomotor Activity in the Open Field in Mice

The protocol for assessing the locomotor response to MK-801 was adapted from Meyer et al. (2008). Mice were injected with saline (0.9% NaCl) or taurine (50, 100, or 200 mg/kg, i.p.) and then placed in the center of an open field arena (40 × 40 × 40 cm) for baseline activity measurement during 30 minutes. Animals were then briefly removed to receive an i.p. injection of MK-801 (0.15 mg/kg), returned to the open field, and locomotor activity was recorded for 60 minutes. The distance traveled in segments of 5 minutes was automatically scored using ANY-Maze software (Stoelting Co., Wood Dale, IL, USA).

#### Prepulse Inhibition (PPI) of the Acoustic Startle Reflex in Mice

Sensorimotor gating was assessed by measuring the PPI of the acoustic startle reflex, which refers to the attenuation of the reaction to a startling stimulus (pulse) when it is shortly preceded by a weaker stimulus (prepulse). The protocol was adapted based on the methodology fully described elsewhere (Meyer et al., 2005). The apparatus consisted of 2 sound-attenuated startle chambers (Insight, Ribeirão Preto, Brazil) equipped with a movement-sensitive platform above which an acrylic enclosure was placed. One day prior to the experiment, subjects were habituated for 10 minutes to the apparatus with background noise (65 dB_A_). On the experiment day, animals received an i.p. injection of either saline (0.9% NaCl) or taurine (50, 100, or 200 mg/kg), followed 30 minutes later by an i.p. injection of saline or MK-801 (0.15 mg/kg). Testing started 30 minutes after the last drug administration. During a 45-minute session, animals were presented to a series of stimuli comprising a mixture of 4 trial types: pulse-alone, prepulse-plus-pulse, prepulse-alone, and no-stimulus (background noise, 65 dB_A_). The startle program consisted of 3 different intensities of a 40-ms white noise pulse (100, 110, and 120 dB_A_) combined or not with 3 different intensities of a 20-millisecond prepulse (71, 77, and 83 dB_A_, which corresponded to 6, 12, and 18 dB above background, respectively). The stimulus-onset asynchrony of the prepulse and pulse stimuli on all prepulse-plus-pulse trials was 100 milliseconds (onset-to-onset). Each session began with a 2-minute acclimation period in the enclosure, followed by 6 consecutive pulse-alone trials to habituate and stabilize the startle response. Subsequently, each stimulus was presented 12 times in a pseudorandom order with an average interval between successive trials of 15 ± 5 seconds. The session was concluded with 6 consecutive pulse-alone trials. Boxes were cleaned with water and dried between sessions. For each subject, PPI was indexed as mean percent inhibition of startle response obtained in the prepulse-plus-trials compared with pulse-alone trials by following the expression: [1-(mean reactivity on prepulse-plus-pulse trials/mean reactivity on pulse-alone trials) × 1/100]. The first and last 6 trials were not included in the calculation of percent PPI. In addition to PPI, reactivity to prepulse- and pulse-alone trials were also analyzed.

#### Social Interaction in Mice

The social interaction protocol was adapted from Jeevakumar et al. (2015). Experimental and stimulus mice were isolated for 24 hours prior to testing. On the day of the experiment, mice received an i.p. injection of either saline (0.9% NaCl) or taurine (50, 100, or 200 mg/kg) followed by another i.p. injection of saline or MK-801 (0.15 mg/kg) 30 minutes later. Testing began 30 minutes after the last injection. A stimulus mouse was placed inside a cylindrical custom-built container (20 cm high, steel bars separated by 1 cm, acrylic lid) and then introduced to the home cage of experimental mice for 10 minutes. All sessions were video-recorded and interaction time (defined as sniffing and investigating at close proximity) were scored offline using Behavioral Observation Research Interactive Software (Friard and Gamba, 2016).

#### Social Preference in Zebrafish

—The protocol for the social preference test in zebrafish followed the method described by Benvenutti et al. (2021). Animals were individually exposed to water or taurine solutions at 42, 150, or 400 mg/L in 500-mL beakers containing 200-mL solution for 20 minutes. They were then transferred to another beaker containing either water or MK-801 at 5 µM for another 20 minutes so that exposure duration for pretreatment and treatment were the same. After exposure, animals were placed for 7 minutes in a tank (30 × 10 × 15 cm) flanked by 2 identical tanks (15 × 101 × 3 cm) either empty (neutral stimulus) or containing 10 unknown zebrafish (social stimulus). All 3 tanks were filled with water in standard conditions at a level of 10 cm. The position of the social stimulus (right or left) was counterbalanced throughout the tests. The water in the test tanks was changed between every animal. To assess social behavior, the test apparatus was virtually divided into 3 vertical zones (interaction, middle, and neutral). Animals were habituated to the apparatus for 2 minutes and then analyzed for the last 5 minutes. Videos were recorded from the front view, and time spent in the interaction zone was quantified as a proxy for social preference. Additionally, total distance traveled, number of crossings between the vertical zones of the tank, and immobility time were quantified as secondary locomotor parameters. All outcomes were automatically scored using ANY-Maze software.

### Statistical Analysis

Outliers were defined following the rule of mean ± 2 SDs. This resulted in 5 outliers removed from the PPI test (1 taurine 50/control, 1 taurine 100/control, 2 taurine 50/MK-801, and 1 taurine 100/MK-801), 5 outliers removed from the social interaction test in mice (1 taurine 50/control, 1 taurine 100/control, 1 taurine 200/control, 1 taurine 50/MK-801, and 1 taurine 200/MK-801), and 2 outliers removed from the social preference test in zebrafish (1 control/control and 1 taurine 42/control). No outliers were removed from the hyperlocomotion test (mean distance traveled after MK-801 was the outcome used for the check). Two mice from the social interaction set died of unknown causes after allocation but before testing (1 control/MK-801 and 1 taurine 100/MK-801).

The sample size to detect a 0.5 effect size with 0.95 power and 0.05 alpha was calculated using Minitab (version 21.1) for Windows; this resulted in n = 10 for locomotor activity (4 groups) and n = 12 for all other assays (8 groups). GraphPad Prism 8 (version 8.4.3) for macOS was used to conduct the statistical analyses and plot the results. For locomotor activity, distance traveled as a function of 5-minute time segments was analyzed using repeated-measures ANOVA, with time as the within-subjects factor and taurine pretreatment as the between-subjects factor; the 2 phases of the experiment (i.e., baseline and MK-801 treatment) were analyzed separately. The data from the remaining experiments were analyzed by 2-way ANOVA, with taurine pretreatment and MK-801 treatment as the main factors. Bonferroni post hoc test was applied as appropriate. The significance level was set at *P *< .05. Data were expressed as mean ± SD.

## RESULTS

### Locomotor Activity in Response to MK-801

**Figure 2.**
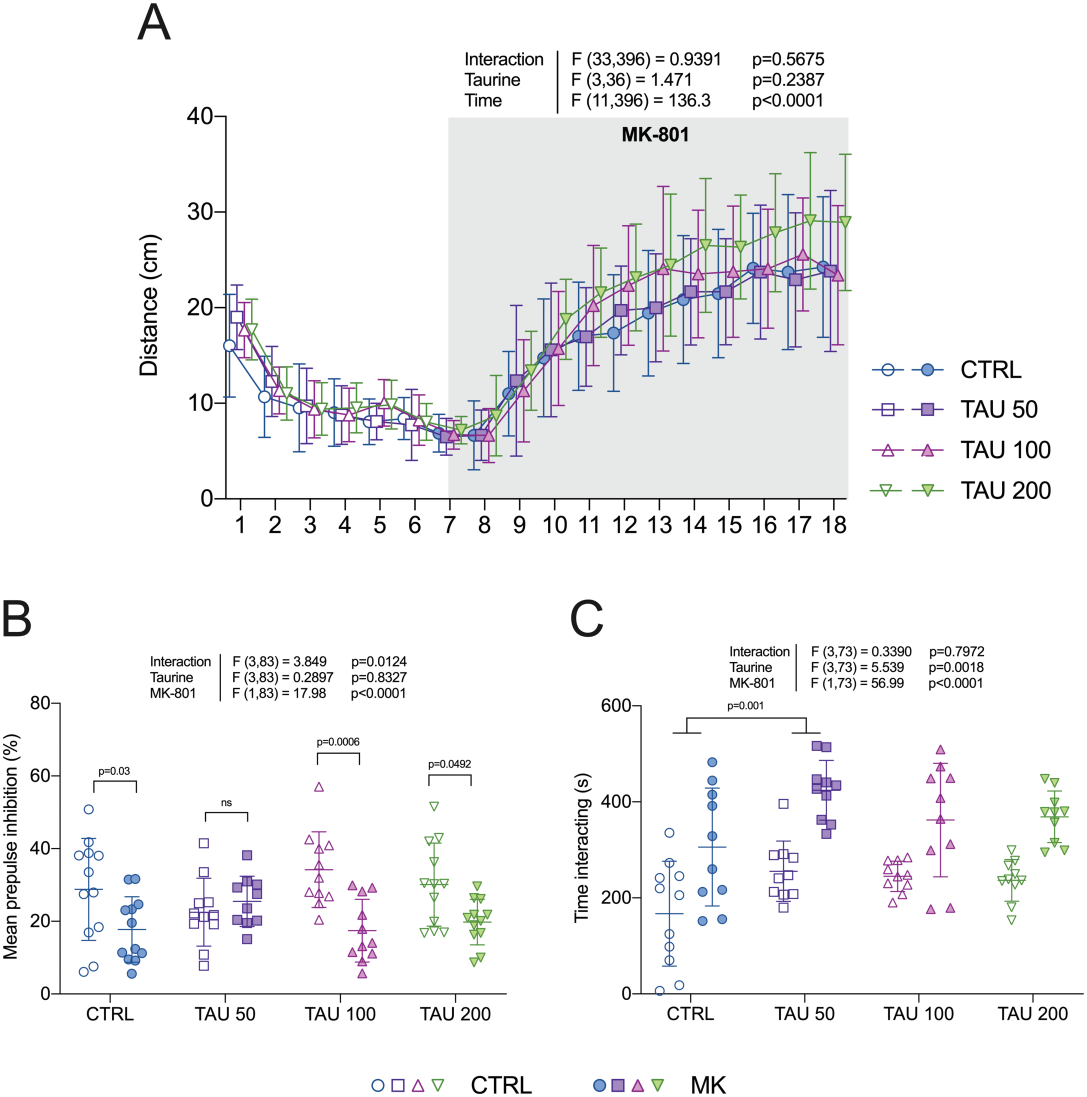
Effects of taurine on behavioral abnormalities induced by MK-801 in mice. (A) Locomotor activity (in segments of 5 minutes), (B) prepulse inhibition of the startle reflex, and (C) social interaction were evaluated as measures relevant to the positive, cognitive, and negative symptoms of schizophrenia, respectively. Two-way ANOVA followed by Bonferroni post-hoc test. Data are presented as mean ± SD, n = 10–12. CTRL = control; TAU = taurine (doses are denoted in mg/kg).

The hyperlocomotion in response to an MK-801 challenge was assessed as an outcome related to the positive symptoms of schizophrenia (Powell and Geyer, 2007). Figure 2A shows that distance travelled by the mice in the open field increased after the MK-801 challenge in all experimental groups (time effect: F_11,396_ = 136.3, *P* < .0001). Taurine did not prevent the effects of MK-801 (taurine effect: F_3,36_ = 1.471, *P* = .2387; interaction effect: F_33,396_ = 0.9391, *P* = .5675). In the baseline phase (first 30 minutes), locomotion decreased as animals habituated to the environment (time effect: F_5,180_ = 102.0, *P* < .0001), and no differences between the groups were observed (taurine effect: F_3,36_ = 0.1631, *P* = .9205; interaction effect: F_15,180_ = 1.140, *P* = .3239).

### MK-801–Induced PPI Deficits

The effects of MK-801 on sensorimotor gating were assessed by the paradigm of PPI of the acoustic startle reflex, which is a translational measure related to the cognitive symptoms of schizophrenia (Powell et al., 2009). Mice treated with MK-801 showed lower levels of PPI compared with controls (MK-801 main effect: F_1,83_ = 17.98, *P* < .0001), indicating deficits in sensorimotor gating (Figure 2B). Two-way ANOVA also revealed a significant interaction effect (F_3,83_ = 3.849, *P* = .0124) in the absence of a taurine main effect (F_3,83_ = 0.2897, *P* = .8327). Bonferroni post hoc tests comparing MK-801 groups with their respective controls resulted in significant differences for all comparisons except for the groups pretreated with taurine at 50 mg/kg (lowest dose), indicating an attenuation of the PPI deficit induced by MK-801.

As expected, reactivity to prepulse-alone trials increased along with prepulse intensity, leading to a main effect of prepulse intensity (F_2,166_ = 92.251, *P* < .01; supplementary Figure 1A). Likewise, a significant main effect was observed for startle intensity when analyzing pulse-alone trials (F_2,166_ = 4.338, *P* = .015; supplementary Figure 1B). A significant MK-801 main effect was also observed for prepulse- (F_1,83_ = 6.003, *P* = .015) and pulse-alone trials (F_1,83_ = 8.024, *P* = .006). Despite the higher reactivity induced by MK-801, a true PPI deficit was supported by analyzing it as absolute reactivity scores in addition to the relative indexation (supplementary Figure 1C); a significant interaction between MK-801 and prepulse intensity was observed (F_3,267_ = 4.991, *P* = .0022), indicating a genuine PPI deficit in animals treated with the NMDA antagonist.

### Social Interaction in Mice

The social behavior toward an unfamiliar mouse introduced in the home cage was evaluated as a phenotype relevant to the negative symptoms of schizophrenia (Jones et al., 2011). Subject mice were manually scored according to their interest in sniffing or investigating at proximity the enclosed stimulus mouse. Figure 2C shows that groups treated with MK-801 spent more time interacting with the social stimulus (MK-801 main effect: F_1,73_ = 56.99, *P* < .0001). Two-way ANOVA also revealed a taurine main effect (F_3,73_ = 5.539, *P* = .0018) without a significant interaction (F_3,73_ = 0.339, *P* = .7972). Bonferroni post hoc comparisons restricted to the pretreatment factor (taurine main effect) indicated that interaction time was significantly higher in groups pretreated with taurine at 50 mg/kg compared with control groups (*P* = .001).

### Social Preference in Zebrafish

**Figure 3.**
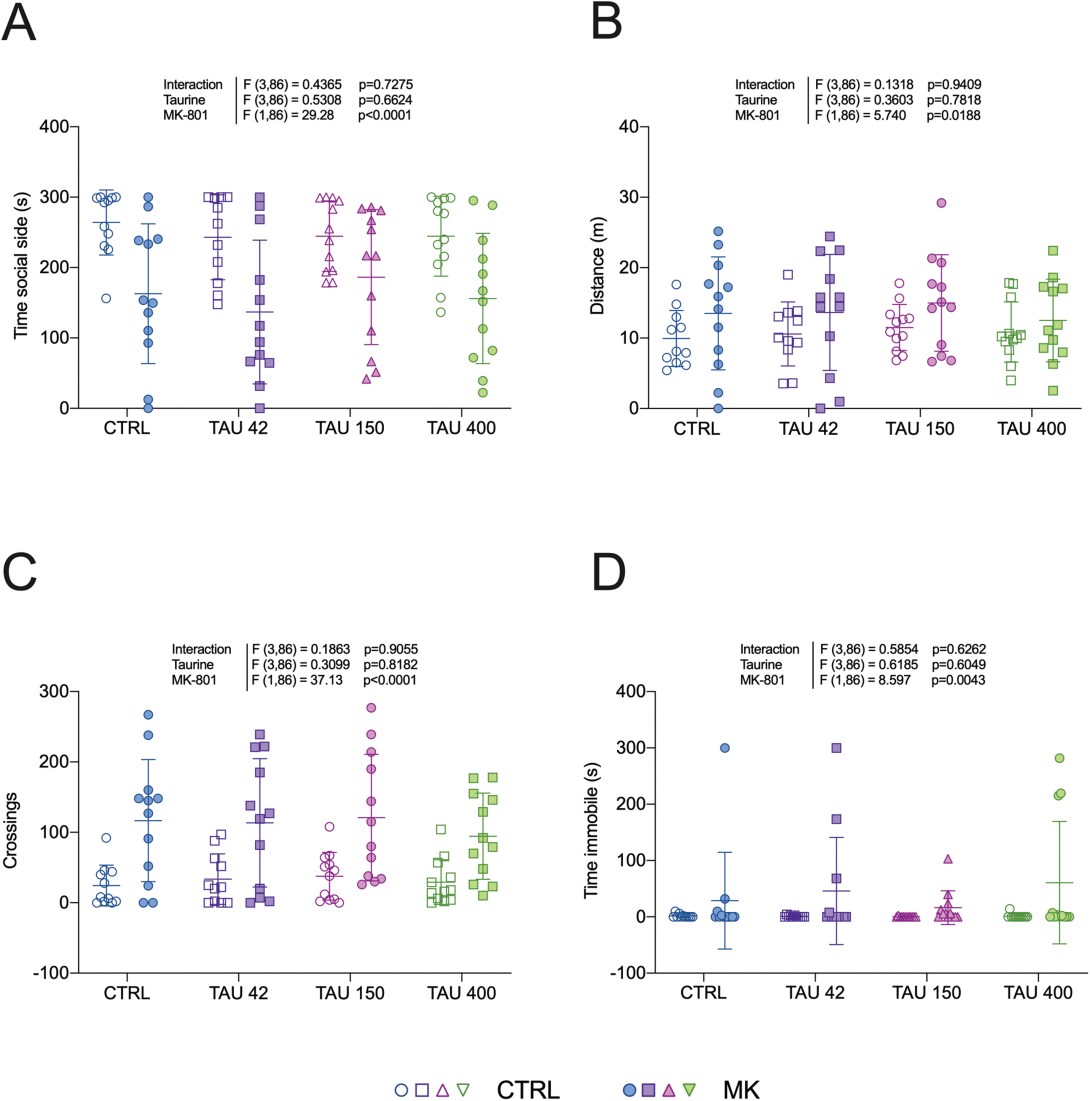
Effects of taurine in the social preference test in zebrafish. (A) Time spent in the social stimulus side was measured as a proxy for social preference, while (B) total distance traveled, (C) number of crossings, and (D) time spent immobile were quantified as secondary locomotor parameters. Two-way ANOVA. Data are presented as mean ± SD, n = 11–12. CTRL = control; TAU = taurine (doses are denoted in mg/L).

Zebrafish is increasingly considered as a model organism suitable to study drug-induced behavioral phenotypes relevant to schizophrenia (Gawel et al., 2019; Benvenutti et al., 2021). Figure 3A shows that zebrafish exposed to MK-801 spent less time in the side of the tank where conspecifics were presented, denoting decreased social preference compared with control groups (MK-801 main effect: F_1,86_ = 29.28, *P* < .0001). The other outcomes evaluated in this test were also altered by MK-801, as shown by increases in total distance travelled (F_1,86_ = 5.74, *P* = .0188; Figure 3B), number of crossings between tank zones (F_1,86_ = 37.13, *P* < .0001; Figure 3C), and immobility time (F_1,86_ = 8.597, *P* = .0043; Figure 3D). Taurine was devoid of effects in all parameters, because no main effects for drug pretreatment or interaction effects were observed.

## DISCUSSION

Animal models provide a unique opportunity to understand how genetic, molecular, and environmental factors might lead to the development of schizophrenia. In this study, we used MK-801 to acutely induce behavioral alterations of translational relevance to schizophrenia in C57BL/6 mice and zebrafish and ultimately evaluate the preventive effects of taurine against the deficits caused by NMDA antagonism in a 2-species approach.

Taurine modulatory activity has been studied in several conditions, including depression, cardiac failure, retina degeneration, and growth problems (Lourenço and Camilo, 2002; Caletti et al., 2018). Here, we found that taurine largely failed to prevent MK-801–induced behavioral alterations. Although a significant interaction was found in the PPI test and post-hoc analysis showed that the group pretreated with taurine at 50 mg/kg before MK-801 administration did not significantly differ from its respective control, this should be interpreted with caution because taurine at this dose seems to buffer PPI to intermediate levels instead of fully preventing the deficit induced by MK-801. In the social interaction test in mice, both groups treated with taurine at 50 mg/kg spent more time interacting compared with pretreatment controls. This agrees with a previous study in which taurine at a similar dose (42 mg/kg) was shown to increase social interaction in Wistar rats (Kong et al., 2006); such increases in social interaction may be explained by the anxiolytic effects reported for taurine in several studies (Kong et al., 2006; El Idrissi et al., 2009; Mezzomo et al., 2016, 2019; Jung and Kim, 2019; Neuwirth et al., 2019; Fontana et al., 2020).

As expected, MK-801 increased the total distance traveled and caused a PPI deficit in mice. In zebrafish, we observed reduced time in the social side as well as hyperlocomotion in all groups exposed to MK-801. Curiously, mice treated with MK-801 spent more time interacting with the stimulus mice compared with controls. This was unexpected because in most studies NMDA antagonism leads to decreased levels of social interaction (Morales and Spear, 2014; Zoicas and Kornhuber, 2019). Jeevakumar et al. (2015), for example, used a similar home cage protocol and observed a significantly reduced investigation time in adult mice exposed to ketamine in the second postnatal week. Although both ketamine and MK-801 are NMDA antagonists, differences might be related to MK-801 being a more specific NMDA antagonist, and ketamine also interacts with dopaminergic and serotoninergic systems (Kapur and Seeman, 2002; Stone et al., 2007). Drug administration regimen and protocol adaptations also might contribute to this difference in social behavior because in our protocol MK-801 was administered acutely in adult animals and social interaction was quantified for 10 continuous minutes, whereas Jeevakumar et al. (2015) assessed social interaction in 5 one-minute consecutive trials following perinatal exposure to ketamine. Moreover, MK-801 showed a fast-acting but unsustainable antidepressant response in control mice (Autry et al., 2011; Zanos et al., 2016), which could explain the increased social behavior in our experiment because mice were tested 30 minutes after the MK-801 injection.

It is well known that excessive stimulation of glutamatergic receptors causes excitotoxicity due to increased intracellular levels of calcium. Previous studies have demonstrated that taurine may act as a neuroprotector either by decreasing intracellular free calcium or by counterbalancing glutamatergic transmission via voltage-gated calcium channels (Lidsky et al., 1995; El Idrissi and Trenkner, 1999; Saransaari and Oja, 2000). Acamprosate, a synthetic analog of taurine, is hypothesized to decrease NMDA receptor activity by modulating the expression of NMDA receptor subunits in specific brain regions (Rammes et al., 2001; Heilig and Egli, 2006). In addition, Chan et al (2015) showed that taurine binds to GluN2B subunit of the NMDA receptor and causes a prolonged inhibition of excitatory synaptic transmission in an ex vivo model.

Although taurine was not able to prevent the deficits observed in our study, it still might have beneficial effects in psychosis models that better mimic the course of schizophrenia, such as neurodevelopmental models. Various studies linked behavioral and neurobiological dysfunctions of schizophrenia to neurodevelopment, which translates into symptoms that appear mainly during late adolescence (Brown, 2006; Knuesel et al., 2014; Volk and Lewis, 2014; Hantsoo et al., 2019). Interventions that aim to act in the prodrome period, preventing the first psychotic episode, are thought to have better outcomes than antipsychotic treatment once they have been ultimately unsuccessful in preventing disease onset in individuals with schizophrenia (McGlashan et al., 2003; McGorry et al., 2013; Woods et al., 2017). Therefore, continuous taurine administration in vulnerability periods might prevent the abnormalities that emerge in early adulthood. With antioxidant and neuroprotector properties, taurine might be able to normalize the altered redox state and parvalbumin-positive interneuron loss found in animal models and patients with schizophrenia (Fung et al., 2010; Gill and Grace, 2014; Salim, 2014; Steullet et al., 2017; Kaar et al., 2019; Goh et al., 2022). Grace et al. (2016) hypothesized that the dysfunction of the dopaminergic system might be a consequence of the loss of many fast-spiking parvalbumin-positive GABAergic interneurons in the ventral subiculum of the hippocampus, causing hyperactivation and dysrhythmic behavior of pyramidal neurons. Taurine might ameliorate this hyperactivation by compensating the inhibitory loss at parvalbumin-positive interneurons and preventing the onset of symptoms in a neurodevelopmental model. Because this dysregulation is postulated to occur in late adolescence or early adulthood, taurine should be administered prior to this period to prevent the disruption of basolateral amygdala, nucleus accumbens, and prefrontal cortex activity and rhythmicity, all of which participate in circuits interconnected with the ventral subiculum.

Regarding zebrafish, it has been demonstrated that MK-801 induces hyperlocomotion, although it is not clear which neuronal mechanisms might be involved (Menezes et al., 2015; Tran et al., 2016; Benvenutti et al., 2021; Franscescon et al., 2021). Increasing use of zebrafish is a great solution to avoid species biases and focus on a robust cross-species approach, aside from being an accessible way to screen for potential novel treatments (Bruni et al., 2016; Burrows and Hannan, 2016; Gawel et al., 2019). Taurine has been demonstrated to prevent MK-801 hyperlocomotion and memory impairment in zebrafish (Franscescon et al., 2020, 2021), a finding that we could not replicate in our study. Here, taurine was not able to counteract MK-801 effects on locomotor activity or social preference. In our protocol, zebrafish were exposed to taurine and MK-801 in a beaker for 20 minutes, and time spent on the social side and distance traveled were assessed. Differences in drug administration route and exposure time might contribute to the divergent outcomes. Whereas i.p. injection in zebrafish is a good alternative to administer drugs that are water insoluble, expensive, or of limited availability, that is not the case for MK-801 and taurine. Because i.p. injection is also a more stressful administration route that requires zebrafish to be anesthetized, we opted for water exposure as the most common and easy route considering species and age (Benvenutti et al., 2022). The only advantage of i.p. injecting zebrafish would be to deliver more precise doses; however, given the small volumes administered and considering that precision syringes are not always employed, that is not necessarily the case for the published studies.

Considering that we also observed a lack of a clear antipsychotic effect of taurine in rodents, we reckon that our findings are robust and consistent across species, which does not necessarily rule out a taurine antipsychotic effect in other treatment regimens. A limitation of our study is that it remains to be established whether taurine can prevent the neuropathological events of schizophrenia in preclinical models that better simulate the course of the disease. Our study was not designed to act in the prodromal phase of schizophrenia, which we believe is a key opportunity window to prevent the alterations that emerge in early adulthood. Another limitation is that preclinical models of schizophrenia likely do not reflect the neurobiology underlying the positive symptoms of the disease, making it difficult to be accurately assessed in behavior tests (Kesby et al., 2018). Because hallucinations are false percepts subjectively perceived as true, a valid assessment of this behavior in rodents can require extensive training, and thus rodents are incompatible with time-sensitive analysis, such as MK-801 acute administration (Schmack et al., 2021). Therefore, other rodent preclinical models of schizophrenia that have a more long-lasting endophenotype, and wherefore allow this type of assessment, may appraise positive-like symptoms with a better predictive validity than the locomotory response to MK-801. Lastly, the use of only male animals in preclinical studies, especially in neuroscience, is a topic that has gained attention lately (Beery and Zucker, 2011). Although we used both male and female zebrafish (because the standard for this species is mixed-sex housing), a limitation of our study is that females were not included in the mouse experiments. However, the use of 2 species (and both sexes for one of them) at least partially compensates this limitation in terms of generalizability.

The frequent failure in translating preclinical findings to clinical settings has been increasingly discussed, and strategies to overcome this loss in translation have been suggested (Seyhan, 2019). The strength of our study lies in including 2 model organisms from different phylogenetic classes, which increases the external validity of preclinical studies. Though more studies are necessary to evaluate taurine’s role in schizophrenia, our 2-species approach contradicts previous studies by showing that, at least acutely and in the doses used in this study, taurine is not able to prevent the behavioral alterations induced by antagonism of NMDA receptors.

## Supplementary Material

pyac073_suppl_Supplementary_Material
