# Supplementary material for: Effects of Taurine in Mice and Zebrafish Behavioral Assays With Translational Relevance to Schizophrenia
Source: Int J Neuropsychopharmacol. 2022 Oct 14;26(2):125–36. doi: 10.1093/ijnp/pyac073 (PMC9926054; doi:10.1093/ijnp/pyac073)
Supplement: pyac073_suppl_Supplementary_Material [file pyac073_suppl_supplementary_material.docx]

# **Supplementary material**

# **Effects of taurine in mice and zebrafish behavioral assays with translational relevance to schizophrenia**

Franciele Kich Giongo, Matheus Gallas-Lopes, Radharani Benvenutti, Adrieli Sachett, Leonardo Marensi Bastos, Adriane Ribeiro Rosa, Ana Paula Herrmann

**Supplementary Figure S1**


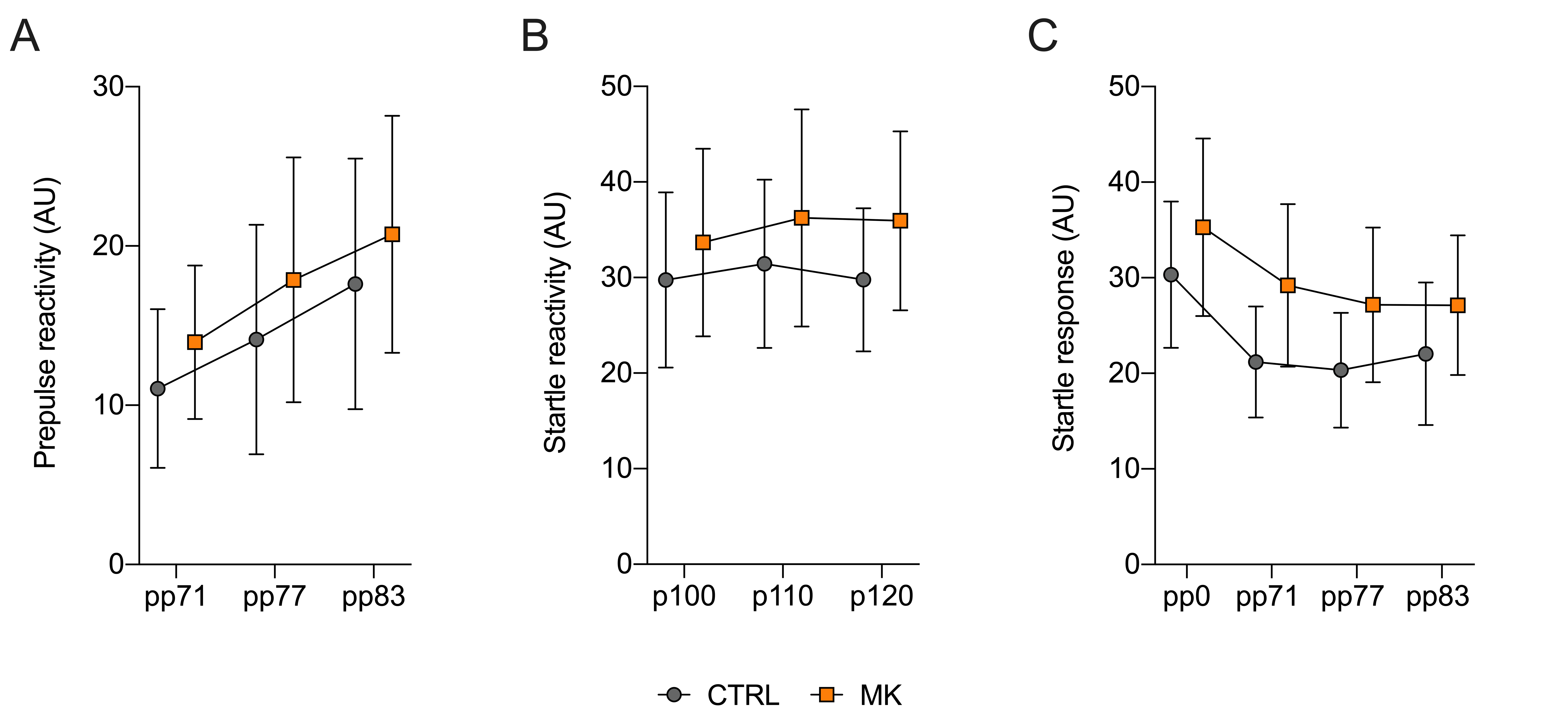


**Figure S1**. Effects of MK-801 on (A) prepulse-induced reactivity, (B) startle reactivity, and (C) prepulse inhibition as indexed by absolute reactivity scores. Two-way ANOVA. Main effect of prepulse intensity (F_2,166_ = 92.251, p<0.01) and MK-801 (F_1,83_ = 6.003, p=0.015) on prepulse-alone trials; main effect of startle intensity (F_2,166_ = 4.338, p=0.015) and MK-801 (F_1,83_ = 8.024, p=0.006) on pulse-alone trials; interaction effect between MK-801 and prepulse intensity on PPI indexed as absolute scores (F_3,267_ = 4.991, p=0.0022). Data are presented as mean ± standard deviation. n=10-12. CTRL: control, MK: MK-801.
